# An Involuntary Proving of Semen Tiglii

**Published:** 1889-08

**Authors:** 


### AN INVOLUNTARY PROVING OF SEMEN TIGLII.

Professor Hugo Schuby narrates, in the *Therap. Monatscheste*, February, '89, that one of his students, when semina tiglii were handed round, swallowed a small particle, about 0.06 grm., containing about 50 per cent. fatty oil. It was about eight A. M. when this robust young man took the drug; at first the taste was not disagreeable, but after a few minutes the taste was that of a mouldy walnut, when he spat out the whole of it. After ten minutes, during which he steadily made efforts to swallow, he felt burning and scratching at the posterior part of the tongue, and all down the pharynx, with a sensation of heat. In about fifteen to twenty minutes that sensation extended down the œsophagus to the stomach. Severe unbearable pains of a drawing character now in the empty stomach—as he had not yet taken breakfast—with nausea and cold sweat on forehead. Intestinal peristalsis strongly increased, and about nine A. M. severe colic, with constant desire to defecate. In going home he had to stop at a tavern to relieve himself; defecation rapid, watery. To remove the burning sensation in the throat he ordered a light breakfast, but could not eat it, as he had to hurry to the closet. Going home he had to stop again at another place, and during the forenoon he had about a dozen discharges.—A. L. Z., May, 1889. S. L.

